# A phase 3 multicenter open-label maintenance study to investigate the long-term safety of sodium zirconium cyclosilicate in Japanese subjects with hyperkalemia

**DOI:** 10.1007/s10157-020-01972-y

**Published:** 2020-10-24

**Authors:** Naoki Kashihara, Yoshimitsu Yamasaki, Takeshi Osonoi, Hiromasa Harada, Yugo Shibagaki, June Zhao, Hyosung Kim, Toshitaka Yajima, Nobuaki Sarai

**Affiliations:** 1 Department of Nephrology and Hypertension, Kawasaki Medical School, Okayama, Japan; 2 AMC Nishi-Umeda Clinic, Osaka, Japan; 3 Department of Internal Medicine, Nakakinen Clinic, Ibaraki, Japan; 4 Departments of Internal Medicine, Yao Tokushukai General Hospital, Osaka, Japan; 5 Division of Nephrology and Hypertension, St. Marianna University School of Medicine Hospital, Kanagawa, Japan; 6 CVRM Late-Stage Development, AstraZeneca Gaithersburg, Gaithersburg, USA; 7 Research and Development, AstraZeneca K.K, 1-8-3, Marunouchi, Chiyoda-ku, Tokyo 100-0005 Japan

**Keywords:** Hyperkalemia, Japanese, Long-term safety study, Sodium zirconium cyclosilicate

## Abstract

**Background:**

Hyperkalemia is associated with many chronic diseases and renin-angiotensin-aldosterone system inhibitor therapy. Sodium zirconium cyclosilicate (SZC), an oral, highly selective cation-exchanger, is approved for the treatment of hyperkalemia.

**Methods:**

This phase 3, multicenter, open-label, single-arm, flexible-dose study assessed the safety and efficacy of SZC in Japanese patients with hyperkalemia during a correction phase of up to 3 days and long-term (1 year) maintenance phase (NCT03172702).

**Results:**

Overall, 150 patients received treatment during both study phases; the study population was generally representative of hyperkalemic Japanese patients in clinical practice. Most patients (78.7%) had three doses of SZC during the correction phase. All but one patient received SZC for ≤ 48 h before transitioning to the maintenance phase. In the maintenance phase, mean (standard deviation; SD) exposure to the study drug was 319.4 (98.1) days and mean (SD) dose was 7.38 (2.85) g/day. Adverse events (AEs) were reported in 131 patients (87.3%); most were mild. The most common treatment-related AEs as evaluated by investigators were constipation (6.7%), peripheral edema (4.0%), and hypertension (2.7%). In the correction phase, 78.7% of patients were normokalemic at 24 h and 98.7% within 48 h; ≥ 65.5% maintained normokalemia throughout the maintenance phase.

**Conclusion:**

After a year of exposure, SZC treatment was well tolerated by Japanese patients and potassium levels were well controlled.

**Supplementary Information:**

The online version contains supplementary material available at 10.1007/s10157-020-01972-y.

## Introduction

Hyperkalemia is an important electrolyte disturbance that is prevalent in patients with many common and chronic diseases, including diabetes mellitus (DM), chronic kidney disease (CKD), and heart failure (HF) [1], and in those using renin-angiotensin-aldosterone system inhibitors (RAASi) [2]. Surprisingly, very few data are available regarding its clinical burden and clinical outcomes. In Japan, a recent observational study using insurance claims data of patients ≥ 18 years old with at least one serum potassium measurement during the study period (mean age 72.4 years; men 55.0%; serum potassium level at index date, 5.4 mEq/L) reported a prevalence of hyperkalemia of 67.9 per 1000 population [3]. Prevalence increased in patients with CKD and HF, and in those using RAASi. Further, an increased risk of death was observed at serum potassium levels of 5.1–5.4 mEq (hazard ratio of 7.6), and hyperkalemia increased the 3 year mortality rates among patients with moderate CKD [3]. Reportedly, 12.3% of 986 Japanese patients with normokalemic CKD who were treated with RAASi during their hospitalization developed hyperkalemia early after discharge [4].

Although there is a need for therapies to treat hyperkalemia that are effective and safe, current treatment options are limited. Despite attempts to control hyperkalemia with dietary restrictions and the use of resins like sodium/calcium polystyrene sulfonate, these treatments either have poor adherence or are limited by uncertain efficacy and potentially serious side effects [5–7].

Sodium zirconium cyclosilicate (SZC) is an oral, non-polymer, highly selective, inorganic cation-exchanger that captures potassium ions in exchange for sodium and hydrogen ions in the gastrointestinal tract, thereby reducing serum potassium concentration in as little as 1 h after administration and removing potassium from the body through increased fecal excretion [8]. SZC has a > 25-fold selectivity for monovalent K^+^ over divalent cations (Ca^2+^ or Mg^2+^) [9]. SZC acts locally and is not absorbed systemically [8]; therefore, SZC is not associated with systemic toxicity [10–12]. In a multicenter, phase 2/3, dose-finding study in 103 Japanese subjects with hyperkalemia (NCT03127644), SZC dosed at 5 g and 10 g three times daily significantly reduced serum potassium levels within 48 h versus placebo and had a good safety and tolerability profile [13]. Moreover, in a subgroup analysis of the same study, consistent beneficial effects of SZC on potassium levels during the correction phase of the study were demonstrated, regardless of underlying condition (HF, DM, CKD, or RAASi treatment) [14]. In the phase 3, multicenter, randomized, double-blind, placebo-controlled HARMONIZE trial, patients with hyperkalemia who were treated with SZC for 28 days achieved normal serum potassium levels within 48 h. All doses of SZC yielded lower potassium levels and higher proportions of patients with normokalemia compared with placebo [12]. Further, SZC was effective regardless of the underlying cause of hyperkalemia, age, sex, race, or baseline potassium level [12]. Similarly, in the phase 3, randomized, double-blind, placebo-controlled HARMONIZE-GLOBAL study of patients with hyperkalemia from 47 sites across Japan, Russia, South Korea, and Taiwan, normal potassium levels were achieved by 63.3% and 89.1% of patients by 24 and 48 h, respectively [15]. Further, the safety profile was similar to that of the HARMONIZE trial, confirming the efficacy and safety of 1 month of SZC administration.

Based on the results of these studies [12–15], and owing to the advantages of SZC over currently available therapies [i.e., rapid potassium-lowering effect, no increase in gastrointestinal adverse events (AEs) or hypomagnesemia, and no clinically meaningful drug–drug interactions [10–12]] SZC has been approved overseas for the treatment of hyperkalemia. However, previous studies have only assessed the short-term efficacy and safety of SZC in Japanese patients. Thus, this study aimed to assess the safety, tolerability, and efficacy of SZC in Japanese patients with hyperkalemia both during the correction phase and during long-term maintenance therapy (1 year administration).

## Methods

### Study design and trial oversight

**Fig. 1:**
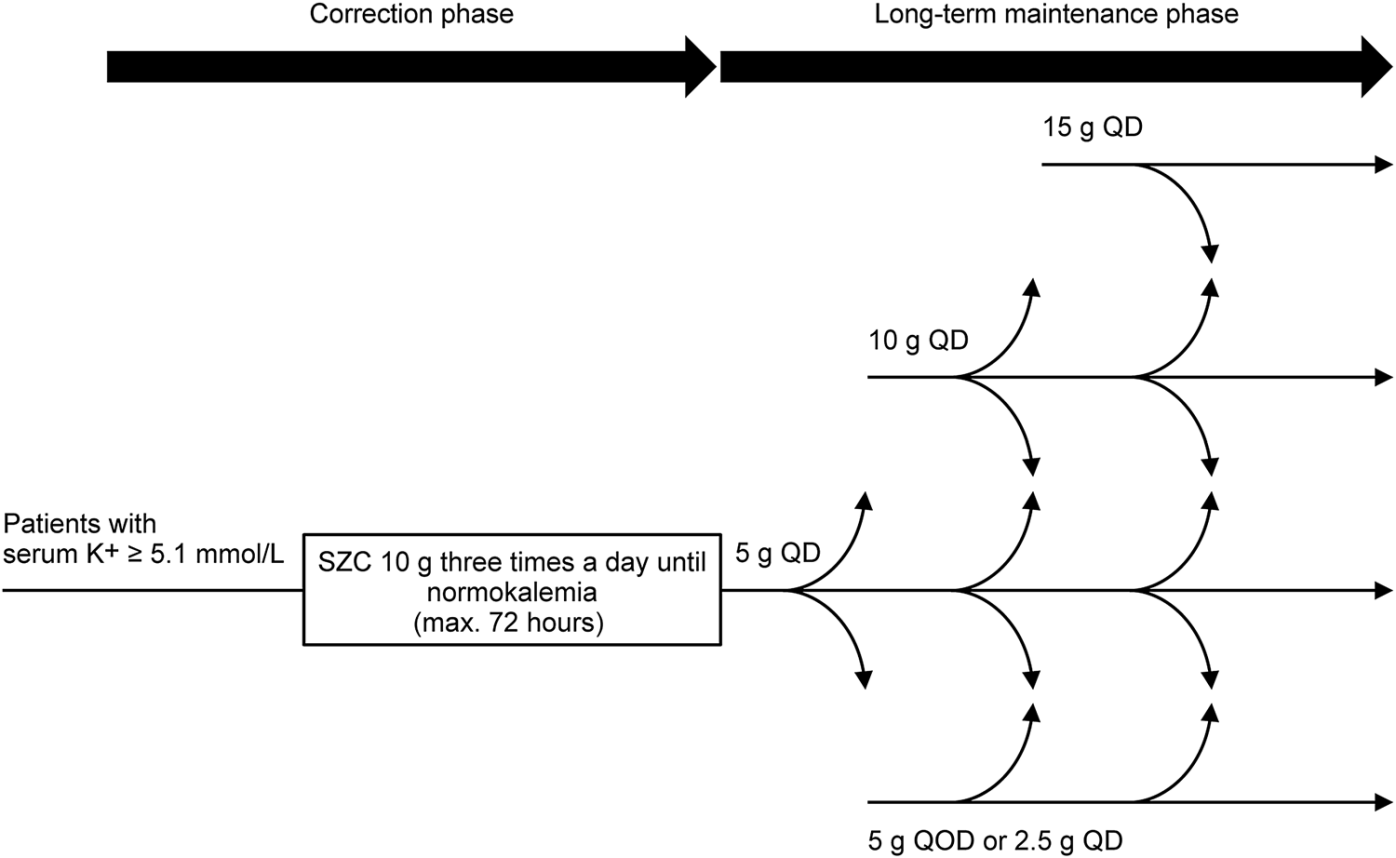
Trial design. *K*^+^ potassium, *Max* maximum, *QD* once daily, *QOD* every other day

This was a phase 3, multicenter, open-label, single-arm, flexible-dose study, comprising a correction phase of up to 3 days, followed by a maintenance phase of up to 1 year (Fig. 1). The institutional review board of each participating institution approved the study protocol and associated documentation. The study was conducted in accordance with the ethical principles originating from the Declaration of Helsinki and are consistent with International Council for Harmonisation/Good Clinical Practice, and applicable regulatory requirements. Informed consent was obtained from each study participant. This study was registered at ClinicalTrials.gov (NCT03172702).

### Patients

The main inclusion criteria were patients aged ≥ 18 years (written informed consent was required from the legal guardian/parent for patients aged < 20 years) with two consecutive potassium values, both ≥ 5.1 mmol/L, using the i-STAT handheld blood analyzer (Abbott Laboratories, Chicago, IL, USA); patients on peritoneal dialysis were eligible if they had a serum potassium level of ≥ 5.5 and ≤ 6.5 mmol/L in two consecutive i-STAT potassium evaluations at least 24 h apart before day 1.

The main exclusion criteria were causes or symptoms of pseudohyperkalemia; treatment with lactulose, rifaxan (rifaximin), or other non-absorbed antibiotics for hyperammonemia within 7 days prior to first SZC dose; treatment with resins such as sevelamer hydrochloride, sodium polystyrene sulfonate (SPS), or calcium polystyrene sulfonate, calcium acetate, calcium carbonate, or lanthanum carbonate, within 7 days prior to first SZC dose; a life expectancy < 12 months; severe physical or mental impairment; female patients who were pregnant; a history of diabetic ketoacidosis; patients with cardiac arrhythmias; hemodialysis patients; and documented glomerular filtration rate (GFR) < 15 mL/min (non-peritoneal dialysis patients only).

### Procedures

The treatment schedules included a correction phase and a maintenance phase. In the correction phase, SZC 10 g was administered three times a day until normokalemia (max 72 h). During the maintenance phase, SZC started at 5 g once daily (QD), was titrated in 5 g increments up to a maximum of 15 g QD or decreased to a minimum of 5 g every other day (QOD; or 2.5 g QD) based on i-STAT potassium measurements.

### Outcomes

The primary outcome measure was safety and tolerability in the maintenance phase. The secondary endpoints were efficacy in the maintenance phase evaluated according to the proportion of patients in whom normokalemia could be maintained for a prolonged period; efficacy and safety in the correction phase; changes in serum aldosterone and bicarbonate during the maintenance phase; and patient-reported outcomes using the Short-Form Health Survey (SF-36) v2. Exploratory endpoints were the evaluation of patient preference of the QOD or QD dose and changes in RAASi use during the study.

### Sample size/rationale

The target sample size was approximately 150 patients, which was the estimated sample size to ensure safety data from ≥ 100 Japanese patients treated with SZC for ≥ 1 year, as required by the International Council for Harmonisation of Technical Requirements for Pharmaceuticals for Human Use (ICH) E1 guidelines [16], and assuming a 35% drop-out rate.

### Statistical methods

The full analysis set (FAS) included all patients who received at least one dose of SZC during the correction and maintenance phases. The safety analysis set (SAS) included all patients in the FAS set, with any safety data.

For the primary endpoint, AEs and AEs of special interest (AESI) were analyzed according to the Medical Dictionary for Regulatory Activities (MedDRA) version 22.0 and summarized descriptively. AESIs were edema-related AEs, cardiac failure, and hypertension. For secondary endpoints, summary statistics were used for efficacy analyses, with a sensitivity analysis for missing serum potassium values. Continuous variables were summarized by descriptive statistics; for categorical variables, frequency and percentage were summarized. No formal statistical testing was performed. The statistical package used was SAS version 9.2 or higher (SAS Institute, Cary, NC, USA).

## Results

### Patient characteristics

**Fig. 2:**
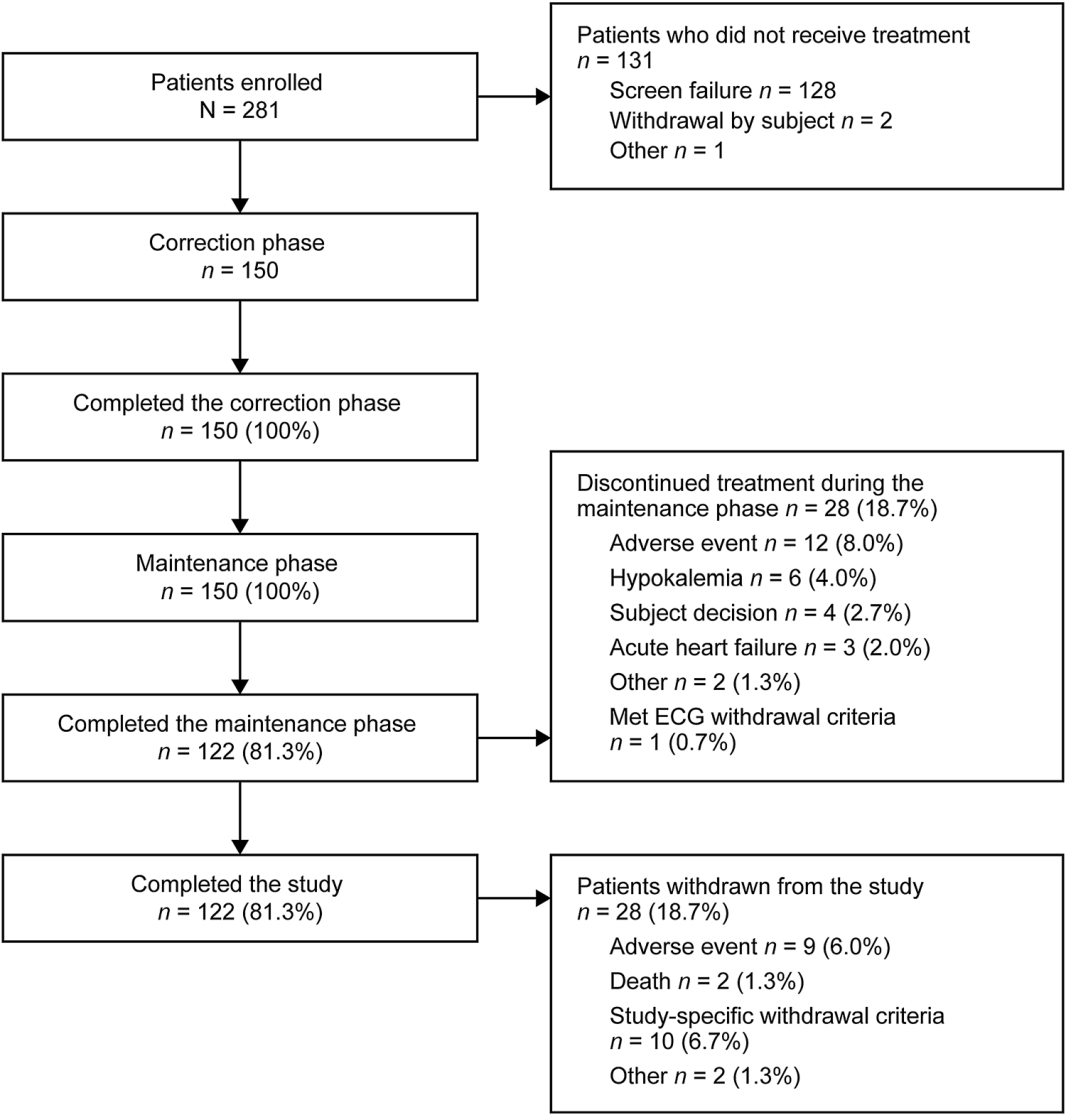
Study flow diagram. *ECG* electrocardiogram

Patients were recruited between September 4, 2017 and July 6, 2019 from 44 centers in Japan. Of 281 subjects enrolled, 131 did not receive treatment (128 failed screening, two subjects withdrew, and one for another reason) and 150 received treatment during the correction phase (Fig. 2). During the correction phase, no patients discontinued treatment; thus, all patients were also treated in the maintenance phase. The FAS and SAS each comprised 150 subjects. In the maintenance phase, 122 patients completed treatment and 28 discontinued. The main reasons for discontinuations were AEs in 12 patients (8.0%), followed by study-specific discontinuation criteria of hypokalemia in six patients (4.0%).

The baseline characteristics of patients in the correction and maintenance phases were the same (Table 1). All patients were of Asian ethnicity, most (74.7%) were men, and the mean [standard deviation (SD)] age was 71.5 (10.8) years. Patients had a mean (SD) body mass index of 24.06 (3.65) kg/m^2^. The mean (SD) estimated GFR (eGFR) was 34.24 (16.46) mL/min/1.73 m^2^. Regarding comorbidities, 14.7% of patients had HF, 58.0% had DM, and 62.7% had CKD. The mean (SD) baseline serum potassium level was 5.70 (0.42) mmol/L. A higher proportion of patients (41.3%) had a baseline serum potassium level between 5.5 and < 6.0 mmol/L, followed by 34.0% with < 5.5 mmol/L, and 24.7% with ≥ 6.0 mmol/L. Most patients in both phases were concomitantly receiving RAASis (71.3%) and 30.0% of patients were receiving diuretics. All patients had a treatment compliance rate between 80 and 120%.

**Table 1:** Baseline demographic characteristics in the safety analysis set

Characteristic	Correction phase (*N* = 150)	Maintenance phase (*N* = 150)
Age (years)		
Mean (SD)	71.5 (10.8)	71.5 (10.8)
Median (range)	73.5 (25–95)	73.5 (25–95)
Age groups (years),* N* (%)		
< 65	25 (16.7)	25 (16.7)
≥ 65	125 (83.3)	125 (83.3)
< 75	82 (54.7)	82 (54.7)
≥ 75	68 (45.3)	68 (45.3)
Sex, *n* (%)		
Male	112 (74.7)	112 (74.7)
Female	38 (25.3)	38 (25.3)
Ethnicity,* N* (%)		
Asian	150 (100.0%)	150 (100.0%)
Height (cm)		
Mean (SD)	161.3 (8.6)	161.3 (8.6)
Weight (kg)		
Mean (SD)	62.8 (11.5)	62.8 (11.5)
Body mass index (kg/m^2^)		
Mean (SD)	24.06 (3.65)	24.06 (3.65)
Serum potassium (mmol/L), *N *(%)		
Mean (SD)	5.70 (0.42)	5.70 (0.42)
< 5.5	51 (34.0)	51 (34.0)
5.5 < 6.0	62 (41.3)	62 (41.3)
≥ 6.0	37 (24.7)	37 (24.7)
eGFR (mL/min/1.73 m^2^),* N* (%)		
Mean (SD)	34.24 (16.46)	34.24 (16.46)
< 15	2 (1.3)	2 (1.3)
15– ≤ 30	73 (48.7)	73 (48.7)
30– ≤ 60	65 (43.3)	65 (43.3)
≥ 60	10 (6.7)	10 (6.7)
Comorbidities, *N* (%)		
HF	22 (14.7)	22 (14.7)
DM	87 (58.0)	87 (58.0)
CKD	94 (62.7)	94 (62.7)
Previous treatments, *N* (%)		
RAAS inhibitor use	107 (71.3)	107 (71.3)
Diuretic use	45 (30.0)	45 (30.0)

*CKD* chronic kidney disease, *DM* diabetes mellitus, *eGFR* estimated glomerular filtration rate, *HF* heart failure, *RAAS* renin-angiotensin-aldosterone system, *SD* standard deviation

### Treatment exposure and safety during the correction phase

The mean (SD) exposure in the correction phase was 1.2 (0.4) days. Most patients (78.7%) had three doses of SZC during the correction phase before moving to the maintenance phase. All but one patient received SZC for ≤ 48 h before transitioning to the maintenance phase.

No AE was reported in more than one patient. Five patients (3.3%) reported AEs (nasopharyngitis, DM, atrioventricular block first degree, hepatic steatosis, and malaise; *n* = 1 each); none were considered causally related to study medication by the investigator. There was only one serious AE (SAE) reported (a case of worsening of DM that was not considered to be causally related to the study treatment; Table 2). No AESIs, or AEs leading to SZC discontinuation or death occurred during this phase. There were no clinically notable changes in mean laboratory values; the incidence of potentially significant clinical abnormalities was low.

**Table 2:** Adverse events in any category during the correction phase and maintenance phase

AE category, *N* (%)	Correction phase (*N* = 150)	Maintenance phase (*N* = 150)
Any AE	5 (3.3)	131 (87.3)
Any AE with death as outcome	0	2 (1.3)
Any SAE (including events with death as outcome)	1 (0.7)	27 (18.0)
Any SAE	0	9 (6.0)
Any AE leading to discontinuation of study drug	0	12 (8.0)
Any causally related AE	0	30 (20.0)
Any AESI		
Edema-related AE	0	31 (20.7)
HF	0	10 (6.7)
Hypertension	0	24 (16.0)

Patients with multiple events in the same category are counted only once in that category. Patients with events in more than one category are counted once in each of those categories

*AE* adverse event, AESI adverse event of special interest, *HF* heart failure, *SAE* serious adverse event

### Treatment exposure during the maintenance phase

During the maintenance phase, the mean (SD) exposure to SZC was 319.4 (98.1) days and the median was 361.0 days. The mean (SD) daily dose of SZC during the maintenance phase was 7.38 (2.85) g/day. The number of exposure days was 358.0 days for the first quartile and 363.0 days for the third quartile. The total patient-years [calculated as the total duration of exposure (days)/365.25] was 131.16.

By the end of the maintenance phase, the total patient-days were 27,160 (56.7%) for SZC 5 g QD, 15,205 (31.7%) for SZC 10 g QD, 4194 (8.8%) for SZC 15 g QD, and 1178 (2.5%) for SZC 2.5 g QD/5 g QOD.

### Safety during the maintenance phase

In the maintenance phase, any AE was reported in 131 patients (87.3%). Two patients (1.3%) had an AE resulting in death (lymphoma and cardiac failure acute; *n* = 1 each; Table 2); neither event was judged to be causally related to the study treatment by the investigator.

SZC was well tolerated over the 12 month maintenance phase. Most AEs were mild. There were no clinically notable mean changes in laboratory values. The incidence of potentially clinically significant abnormalities in laboratory findings was low. The most common AEs were nasopharyngitis (24.0%), hypertension (15.3%), peripheral edema (15.3%), and constipation (15.3%; Table 3). AEs considered by investigators to be causally related to SZC treatment had an overall incidence of 20.0% (*n* = 30). The most common were constipation (6.7%), peripheral edema (4.0%), and hypertension (2.7%).

**Table 3:** Adverse events occurring in ≥ 2% of patients in the maintenance phase (*N* = 150)

Preferred term	Patients^a^ *N* (%)
Patients with any AE	131 (87.3)
Nasopharyngitis	36 (24.0)
Hypertension	23 (15.3)
Peripheral edema	23 (15.3)
Constipation	23 (15.3)
Cataract	7 (4.7)
Generalized edema	7 (4.7)
Arthralgia	6 (4.0)
Back pain	6 (4.0)
Contusion	6 (4.0)
Cough	6 (4.0)
Hypoglycemia	6 (4.0)
Pneumonia	6 (4.0)
Type 2 diabetes mellitus	6 (4.0)
Cardiac failure congestive	5 (3.3)
Hyperuricemia	5 (3.3)
Eczema asteatotic	4 (2.7)
Influenza	4 (2.7)
Nausea	4 (2.7)
Pain in extremity	4 (2.7)
Abdominal upper pain	3 (2.0)
Cardiac failure	3 (2.0)
Chest pain	3 (2.0)
Chronic kidney disease	3 (2.0)
Diabetic retinopathy	3 (2.0)
Diarrhea	3 (2.0)
Dizziness	3 (2.0)
Headache	3 (2.0)
Hyperphosphatemia	3 (2.0)
Nephrogenic anemia	3 (2.0)
Periodontal disease	3 (2.0)
Pharyngitis	3 (2.0)
Tinnitus	3 (2.0)
Vomiting	3 (2.0)

*AE* adverse event

^a^Number (%) of patients with AEs, sorted in decreasing frequency for preferred term in all patients

The overall incidence of SAEs was 18.0%. Congestive HF was reported by four patients (2.7%), pneumonia by three patients (2.0%), and cataract by two patients (1.3%); no other SAEs were reported by > 1 patient. The incidence of AESI were as follows: hypertension [Standardised MedDRA Query (SMQ) “hypertension” (narrow)], 16.0%; edema-related events (MedDRA preferred term “fluid overload”, “fluid retention”, “generalised edema”, “hypervolaemia”, “localised edema”, “edema”, “peripheral edema”, and “peripheral swelling”), 20.7%; and HF (SMQ “cardiac failure” [narrow]), 6.7%. AEs leading to SZC discontinuation occurred in 8.0% of patients. Twenty-nine patients (19.3%) had at least one serum potassium value < 3.5 mmol/L; of these, 3.3% had moderate hypokalemia (≥ 2.5 to < 3.0 mmol/L). No patients developed severe hypokalemia (< 2.5 mmol/L) during the maintenance phase. There was one instance each of electrocardiogram QT prolonged, cardiovascular sudden death (SAE of acute HF), and ventricular fibrillation.

### Serum potassium in the correction phase

In the correction phase, as assessed by i-STAT, the proportions of normokalemic patients were 78.7% (118 patients) at 24 h, 98.7% (148 patients) by or before 48 h, and 99.3% (149 patients) by or before 72 h. By central assessment, the proportions of normokalemic patients were 65.3% (98 patients) at 24 h, 81.3% (122 patients) by or before 48 h, and 82.0% (123 patients) by or before 72 h. Only one patient received SZC treatment for up to 72 h.

### Serum potassium in the maintenance phase

**Fig. 3:**
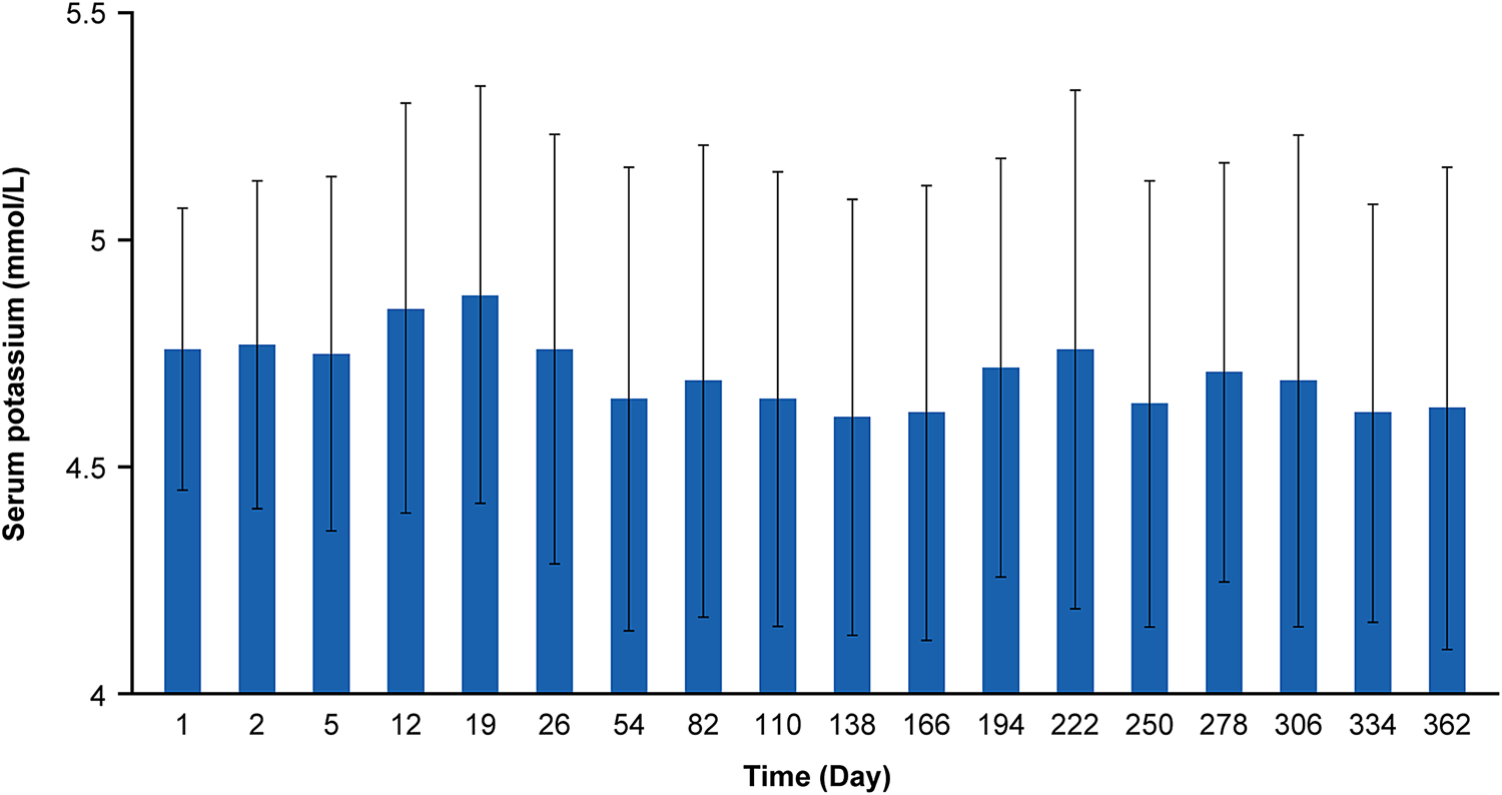
Mean serum potassium (mmol/L) over time (± standard deviation) (full analysis set, maintenance phase)

The serum potassium levels over time are shown in Fig. 3. Most patients (82.0%) were normokalemic on day 1 and maintained normokalemia throughout the maintenance phase. Day 138 showed the highest proportion of normokalemic patients (85.1%) and ≥ 65.5% maintained normokalemia throughout. At Visit 23 (day 362: the last scheduled visit), 79.5% of patients were normokalemic. After the last SZC treatment, the mean (SD) serum potassium level was 4.52 (0.62) mmol/L. Subgroup analyses showed consistent results across subpopulations defined by age, sex, baseline serum potassium, eGFR, weight, RAASi use, diuretic use, and comorbid conditions of DM, HF, and CKD (Supplementary Table 1).

Mean (SD) serum aldosterone at baseline was 104.908 (140.981). A mean (SD) reduction in serum aldosterone of 48.561 (129.261) pmol/L [95% confidence interval (CI) − 71.827– − 25.295] to 58.672 (66.860) pmol/L was observed from baseline to the maintenance phase day 362. At baseline, 99.3% of patients had a serum aldosterone value within the normal range, while on maintenance day 362, all patients still receiving treatment (*n* = 122) were within the normal range for serum aldosterone.

Mean (SD) serum bicarbonate at baseline was 20.92 (2.88) mmol/L. A mean (SD) increase in serum bicarbonate levels of 1.50 (2.85) mmol/L (95% CI 0.99–2.01) to 22.47 (3.15) mmol/L was observed from baseline to maintenance phase day 362. The proportion of patients with serum bicarbonate within the normal range increased from 90.0% at baseline to 98.0% at maintenance phase day 1, and remained between 95.9% and 99.2% for the remainder of the maintenance phase.

Regarding patient-reported outcomes using SF-36, a higher proportion of patients reported better health transition scores post-treatment compared with those at baseline [34 (26.6%) and 27 (18.0%) patients, respectively], and a lower proportion of patients reported worse scores [14 (10.9%) and 32 (21.3%) patients, respectively)]. Of note, post-treatment data were missing for 22 patients.

Exploratory efficacy analyses assessed changes in RAASi use during the study. Most patients did not change their RAASi dose during SZC treatment.

## Discussion

This phase 3, multicenter, open-label, single-arm, flexible-dose study was designed to evaluate the long-term safety, tolerability, and efficacy of SZC after 1 year of administration in Japanese patients with hyperkalemia. The results demonstrated that after a year of exposure, SZC had a positive safety profile, treatment was well-tolerated by Japanese patients and potassium levels were well controlled. Importantly, the Japanese patient population in this study was considered representative of real-world Japanese patients with hyperkalemia undergoing chronic management [3], supporting the applicability of these results to the wider Japanese population.

The key results obtained during the maintenance phase of the present study were that SZC was well tolerated (primary objective), and the tolerability profile was consistent with previous SZC studies in Japan and other Asian countries [13, 15]. The present safety profile was also comparable with that of previous studies conducted overseas [10–12]. Most AEs were mild or moderate in intensity, and not considered causally related to SZC. In total, 18.0% of patients had an SAE and 8.0% of patients discontinued SZC treatment due to an AE.

Regarding the key secondary outcomes, the majority of patients (≥ 65.5%) remained normokalemic throughout the maintenance phase, with the highest proportion (85.1%) on day 138. Importantly, all patients had an average serum potassium level of ≤ 5.5 mmol/L over the entire maintenance phase. Furthermore, subgroup analyses showed consistent results across subpopulations. These findings are consistent with those reported for shorter courses of treatment as in the dose-finding study and HARMONIZE-GLOBAL studies, which included Japanese patients [13, 15]. Thus, the present findings confirm that SZC is effective in inducing and maintaining normokalemia long term, regardless of age, sex, renal function, concomitant medication, or comorbid conditions in Japanese patients.

Changes within the normal range in serum aldosterone and bicarbonate were reported during the maintenance phase. Serum potassium concentration and aldosterone production have a correlative relationship; the observed decrease in serum aldosterone levels following treatment with SZC is consistent with the expected response to a decrease in serum potassium [17]. The mechanism of increased serum bicarbonate following treatment with SZC has not been fully elucidated. With a pore diameter of ~ 3.0 Å for SZC, it is possible that ammonium (2.98 Å), in addition to potassium (2.96 Å), has a high affinity for SZC [9]. Through binding to SZC, ammonium reabsorption from the gastrointestinal tract may be decreased, resulting in reduced urea synthesis and a lower consumption of bicarbonate within the liver [18].

During the correction phase, there was one SAE and no treatment-related AEs or AEs leading to SZC discontinuation, supporting the tolerability of SZC. Hypocalcemia, a common AE following treatment with SPS, was not induced by SZC. This is likely due to the chemical composition and diameter of the micropores of SZC, resulting in the high ion affinity (> 25-fold) for K^+^ over other divalent cations (Ca^2+^ or Mg^2+^). Whereas in mixed ionic media, the selectivity of SPS for K^+^ was 0.2–0.3 times its selectivity for Ca^2+^ or Mg^2+^ [9]. Regarding AESI, rates of HF (6.7%) and edema-related events (20.7%) in the current study were similar to those previously reported in a 12-month, phase 3, dose-titration study of SZC [19], during which 2% of patients had congestive cardiac failure (all of whom discontinued treatment), and 10% had peripheral edema (15% with edema-related events). In that study, patients with edema-related events tended to display characteristics likely to elevate the risk of edema, including renal impairment (eGFR < 45 mL/min/1.73 m^2^), HF, older age, and use of a calcium channel blocker/diuretic at baseline [19]. Given the demographic and clinical characteristics of patients in our study (50.0% had eGFR ≤ 30 mL/min/1.73 m^2^, 14.7% had HF, 45.3% were aged ≥ 75 years, and 30.0% had used diuretics at baseline), the occurrence of edema was not unexpected. Similarly, constipation occurred more frequently during this study than in a similar study of SZC in the non-Japanese population (15.3% and 6%, respectively) [19]. This may reflect the increased number of patients with advanced CKD included within this trial compared with the previous study (50% and 39% of patients with Stage 4 or 5 CKD, respectively).

During the maintenance phases of study ZS-003 (AstraZeneca, data on file) and HARMONIZE [12], exposure-adjusted rates of edema-related treatment-emergent AEs were higher in the SZC group compared with the placebo group. However, in long-term studies (up to 12 months) which allowed titration of SZC to maintain normokalemia, exposure-adjusted rates of edema with SZC treatment were not increased relative to that observed for placebo in studies with shorter maintenance duration (AstraZeneca, data on file). In the 12 month, phase 3, dose-titration study of SZC, SMQ edema was reported more frequently in patients receiving higher SZC doses; this finding can be attributed to the fact that higher SZC doses were more commonly necessary in patients with multiple comorbidities and baseline risk factors [19]. Thus, although the majority of previously reported edema-related events were mild in severity, as in the present study, it would be wise for physicians to monitor patients with factors predisposing towards the development of edema during SZC treatment. No comparative data are available to assess the relative frequency of edema, HF, and hypertension following treatment with SZC versus SPS.

In randomized, double-blind, placebo-controlled studies, dose-related increases in the number of patients with serum potassium values < 3.5 mmol/L were observed during the maintenance phase of SZC treatment (AstraZeneca, data on file). In studies ZS-004E and ZS-005, seven (5.7%) and 49 (6.6%) patients treated with SZC, respectively, had confirmed hypokalemia during the maintenance phase. Although most of these events were mild in severity as in the present study, serious events such as ventricular fibrillation have been reported (AstraZeneca, data on file); thus, careful monitoring and adequate treatment are needed. The risk of clinically significant hypokalemia in subjects treated with SZC is generally low and can be managed by careful monitoring and adequate medical care.

One of the main limitations of this study was the lack of a control group; therefore, there is a limit to determining the relationship between the drug and any observed phenomenon. Additionally, the data analyzed are from Japanese patients only. Therefore, caution is needed when extrapolating to other populations.

In conclusion, over 12 months, SZC demonstrated a good safety profile and was well tolerated in Japanese patients with hyperkalemia (serum potassium ≥ 5.1 mmol/L). Normokalemia was established and maintained with SZC in this patient population. SZC will be a useful option for the treatment of hyperkalemia.

## Supplementary Information

Below is the link to the electronic supplementary material.

Supplementary file1 (DOCX 30 KB)
