# Supplementary material for: A phase 3 multicenter open-label maintenance study to investigate the long-term safety of sodium zirconium cyclosilicate in Japanese subjects with hyperkalemia
Source: Clin Exp Nephrol. 2020 Oct 24;25(2):140–9. doi: 10.1007/s10157-020-01972-y (PMC7880936; doi:10.1007/s10157-020-01972-y)
Supplement: Supplementary file 1 — Supplementary file1 (DOCX 30 KB) [file 10157_2020_1972_MOESM1_ESM.docx]

Supplemental Material

A phase 3 multicenter open-label maintenance study to investigate the long-term safety of sodium zirconium cyclosilicate in Japanese subjects with hyperkalemia

Naoki Kashihara^1^; Yoshimitsu Yamasaki^2^; Takeshi Osonoi^3^; Hiromasa Harada^4^; Yugo Shibagaki^5^; June Zhao^6^; Hyosung Kim^7^; Toshitaka Yajima^7^; Nobuaki Sarai^7^

1. Department of Nephrology and Hypertension, Kawasaki Medical School, Okayama, Japan

2. AMC Nishi-Umeda Clinic, Osaka, Japan

3. Nakakinen Clinic, Ibaraki, Japan

4. Yao Tokushukai General Hospital, Osaka, Japan

5. St. Marianna University School of Medicine Hospital, Kanagawa, Japan

6. AstraZeneca Gaithersburg, Gaithersburg, USA

7. AstraZeneca K.K., Osaka, Japan

**Supplemental Table 1. Proportion of normokalemic patients over time by subgroup, full analysis set**

| **Subgroup category** | | **Time point** | **N** | **Normokalemia**  **(3.5–5.0 mmol/L)** | | |
| --- | --- | --- | --- | --- | --- | --- |
|  |  |  |  | **n** | **%** | **95% CI** |
| Age group 1 (years) | <65 | CP baseline | 25 | 1 | 4.0 | 0.1, 20.4 |
|  |  | MP baseline (Day 1) | 25 | 23 | 92.0 | 74.0, 99.0 |
|  |  | MP Day 362 | 24 | 18 | 75.0 | 53.3, 90.2 |
|  | ≥65 | CP baseline | 125 | 3 | 2.4 | 0.5, 6.9 |
|  |  | MP baseline (Day 1) | 125 | 100 | 80.0 | 71.9, 86.6 |
|  |  | MP Day 362 | 98 | 79 | 80.6 | 71.4, 87.9 |
| Age group 2 (years) | <75 | CP baseline | 82 | 2 | 2.4 | 0.3, 8.5 |
|  |  | MP baseline (Day 1) | 82 | 70 | 85.4 | 75.8, 92.2 |
|  |  | MP Day 362 | 73 | 57 | 78.1 | 66.9, 86.9 |
|  | ≥75 | CP baseline | 68 | 2 | 2.9 | 0.4, 10.2 |
|  |  | MP baseline (Day 1) | 68 | 53 | 77.9 | 66.2, 87.1 |
|  |  | MP Day 362 | 49 | 40 | 81.6 | 68.0, 91.2 |
| Sex | Male | CP baseline | 112 | 2 | 1.8 | 0.2, 6.3 |
|  |  | MP baseline (Day 1) | 112 | 87 | 77.7 | 68.8, 85.0 |
|  |  | MP Day 362 | 91 | 74 | 81.3 | 71.8, 88.7 |
|  | Female | CP baseline | 38 | 2 | 5.3 | 0.6, 17.7 |
|  |  | MP baseline (Day 1) | 38 | 36 | 94.7 | 82.3, 99.4 |
|  |  | MP Day 362 | 31 | 23 | 74.2 | 55.4, 88.1 |
| Weight (kg) | <65 | CP baseline | 91 | 3 | 3.3 | 0.7, 9.3 |
|  |  | MP baseline (Day 1) | 91 | 75 | 82.4 | 73.0, 89.6 |
|  |  | MP Day 362 | 75 | 60 | 80.0 | 69.2, 88.4 |
|  | ≥65 | CP baseline | 59 | 1 | 1.7 | 0.0, 9.1 |
|  |  | MP baseline (Day 1) | 59 | 48 | 81.4 | 69.1, 90.3 |
|  |  | MP Day 362 | 47 | 37 | 78.7 | 64.3, 89.3 |
| Baseline sK^+^ (mmol/L) | <5.5 | CP baseline | 51 | 4 | 7.8 | 2.2, 18.9 |
|  |  | MP baseline (Day 1) | 51 | 43 | 84.3 | 71.4, 93.0 |
|  |  | MP Day 362 | 41 | 34 | 82.9 | 67.9, 92.8 |
|  | 5.5 –<6.0 | CP baseline | 62 | 0 | 0.0 | 0.0, 5.8 |
|  |  | MP baseline (Day 1) | 62 | 52 | 83.9 | 72.3, 92.0 |
|  |  | MP Day 362 | 48 | 40 | 83.3 | 69.8, 92.5 |
|  | ≥6.0 | CP baseline | 37 | 0 | 0.0 | 0.0, 9.5 |
|  |  | MP baseline (Day 1) | 37 | 28 | 75.7 | 58.8, 88.2 |
|  |  | MP Day 362 | 33 | 23 | 69.7 | 51.3, 84.4 |
| Baseline eGFR (mL/min/ 1.73 m^2^) | <30 | CP baseline | 75 | 0 | 0.0 | 0.0, 4.8 |
|  |  | MP baseline (Day 1) | 75 | 59 | 78.7 | 67.7, 87.3 |
|  |  | MP Day 362 | 58 | 45 | 77.6 | 64.7, 87.5 |
|  | 30 – <60 | CP baseline | 65 | 4 | 6.2 | 1.7, 15.0 |
|  |  | MP baseline (Day 1) | 65 | 55 | 84.6 | 73.5, 92.4 |
|  |  | MP Day 362 | 54 | 43 | 79.6 | 66.5, 89.4 |
|  | ≥60 | CP baseline | 10 | 0 | 0.0 | 0.0, 30.8 |
|  |  | MP baseline (Day 1) | 10 | 9 | 90.0 | 55.5, 99.7 |
|  |  | MP Day 362 | 10 | 9 | 90.0 | 55.5, 99.7 |
| RAASi use | Yes | CP baseline | 107 | 2 | 1.9 | 0.2, 6.6 |
|  |  | MP baseline (Day 1) | 107 | 88 | 82.2 | 73.7, 89.0 |
|  |  | MP Day 362 | 81 | 62 | 76.5 | 65.8, 85.2 |
|  | No | CP baseline | 43 | 2 | 4.7 | 0.6, 15.8 |
|  |  | MP baseline (Day 1) | 43 | 35 | 81.4 | 66.6, 91.6 |
|  |  | MP Day 362 | 41 | 35 | 85.4 | 70.8, 94.4 |
| Diuretic use | Yes | CP baseline | 45 | 2 | 4.4 | 0.5, 15.1 |
|  |  | MP baseline (Day 1) | 45 | 36 | 80.0 | 65.4, 90.4 |
|  |  | MP Day 362 | 33 | 28 | 84.8 | 68.1, 94.9 |
|  | No | CP baseline | 105 | 2 | 1.9 | 0.2, 6.7 |
|  |  | MP baseline (Day 1) | 105 | 87 | 82.9 | 74.3, 89.5 |
|  |  | MP Day 362 | 89 | 69 | 77.5 | 67.4, 85.7 |
| Heart failure | Yes | CP baseline | 22 | 1 | 4.5 | 0.1, 22.8 |
|  |  | MP baseline (Day 1) | 22 | 17 | 77.3 | 54.6, 92.2 |
|  |  | MP Day 362 | 18 | 16 | 88.9 | 65.3, 98.6 |
|  | No | CP baseline | 128 | 3 | 2.3 | 0.5, 6.7 |
|  |  | MP baseline (Day 1) | 128 | 106 | 82.8 | 75.1, 88.9 |
|  |  | MP Day 362 | 104 | 81 | 77.9 | 68.7, 85.4 |
| Diabetes | Yes | CP baseline | 87 | 3 | 3.4 | 0.7, 9.7 |
|  |  | MP baseline (Day 1) | 87 | 69 | 79.3 | 69.3, 87.3 |
|  |  | MP Day 362 | 70 | 55 | 78.6 | 67.1, 87.5 |
|  | No | CP baseline | 63 | 1 | 1.6 | 0.0, 8.5 |
|  |  | MP baseline (Day 1) | 63 | 54 | 85.7 | 74.6, 93.3 |
|  |  | MP Day 362 | 52 | 42 | 80.8 | 67.5, 90.4 |
| Chronic kidney disease | Yes | CP baseline | 94 | 2 | 2.1 | 0.3, 7.5 |
|  |  | MP baseline (Day 1) | 94 | 73 | 77.7 | 67.9, 85.6 |
|  |  | MP Day 362 | 74 | 57 | 77.0 | 65.8, 86.0 |
|  | No | CP baseline | 56 | 2 | 3.6 | 0.4, 12.3 |
|  |  | MP baseline (Day 1) | 56 | 50 | 89.3 | 78.1, 96.0 |
|  |  | MP Day 362 | 48 | 40 | 83.3 | 69.8, 92.5 |

CI, confidence interval; CP, correction phase; eGFR, estimated glomerular filtration rate;
MP, maintenance phase; sK^+^, serum potassium concentration
